# An investigation into factors affecting electron density calibration for a megavoltage cone‐beam CT system

**DOI:** 10.1120/jacmp.v13i5.3271

**Published:** 2012-09-06

**Authors:** Jessica Hughes, Lois C. Holloway, Alexandra Quinn, Andrew Fielding

**Affiliations:** ^1^ School of Physical and Chemical Science Queensland University of Technology Brisbane Queensland; ^2^ Department of Physics Liverpool and Macarthur Cancer Therapy Centres New South Wales; ^3^ Department of Physics Premion First in Cancer Care Queensland; ^4^ Institute of Medical Physics University of Sydney New South Wales; ^5^ Centre for Medical Radiation Physics University of Wollongong New South Wales Australia

**Keywords:** electron density, MV cone beam

## Abstract

There is a growing interest in the use of megavoltage cone‐beam computed tomography (MV CBCT) data for radiotherapy treatment planning. To calculate accurate dose distributions, knowledge of the electron density (ED) of the tissues being irradiated is required. In the case of MV CBCT, it is necessary to determine a calibration‐relating CT number to ED, utilizing the photon beam produced for MV CBCT. A number of different parameters can affect this calibration. This study was undertaken on the Siemens MV CBCT system, MVision, to evaluate the effect of the following parameters on the reconstructed CT pixel value to ED calibration: the number of monitor units (MUs) used (5, 8, 15 and 60 MUs), the image reconstruction filter (head and neck, and pelvis), reconstruction matrix size (256 by 256 and 512 by 512), and the addition of extra solid water surrounding the ED phantom. A Gammex electron density CT phantom containing EDs from 0.292 to 1.707 was imaged under each of these conditions. The linear relationship between MV CBCT pixel value and ED was demonstrated for all MU settings and over the range of EDs. Changes in MU number did not dramatically alter the MV CBCT ED calibration. The use of different reconstruction filters was found to affect the MV CBCT ED calibration, as was the addition of solid water surrounding the phantom. Dose distributions from treatment plans calculated with simulated image data from a 15 MU head and neck reconstruction filter MV CBCT image and a MV CBCT ED calibration curve from the image data parameters and a 15 MU pelvis reconstruction filter showed small and clinically insignificant differences. Thus, the use of a single MV CBCT ED calibration curve is unlikely to result in any clinical differences. However, to ensure minimal uncertainties in dose reporting, MV CBCT ED calibration measurements could be carried out using parameter‐specific calibration measurements.

PACS number: 87.59.bd

## I. INTRODUCTION

In radiotherapy, imaging is an increasingly important component of the treatment planning and delivery verification process. It is necessary to ensure the accurate delivery of dose to the target and sparing of normal tissue. With advances in treatment techniques, interest in three‐dimensional (3D) imaging at the time of treatment has increased, as has the possibility of using such images within the planning process.

Cone‐beam computed tomography (CBCT) is one possible mechanism and there are many recent developments of these systems utilizing both kilovoltage (kV)^1^, ^5^ and megavoltage (MV) radiation beams.^6^, ^9^ MV CBCT utilizes the therapy beam and involves the acquisition of a number of two‐dimensional (2D) cone beam projection images from different angles around the patient which are then used to reconstruct a 3D image dataset. This can be used to verify patient setup^10^, ^11^ and volume changes^(12)^ over the course of the radiotherapy treatment, and to facilitate the optimization or adaption of treatment plans over the relatively long course of a treatment.^12^, ^13^ MV CBCT images can also be used to replace or complement the kV CT planning data when there are high‐density objects present, such as prosthesis or dental implants.^(8)^ These objects cause artifacts in kV images which limit visualization of anatomy and the usefulness of heterogeneity corrections.^(14)^ These artifacts, however, have little impact on the image quality of MV CBCT images.^(15)^ The use of daily MV CBCT images for patient setup verification in the presence of orthopedic hardware has also been shown to be possible.^(7)^

To utilize MV CBCT images for optimization or adaption of treatment plans over the course of treatment, or in place of kV CT scans in the initial treatment planning process,^13^, ^16^, ^17^ a reliable calibration between the pixel values (reconstructed gray‐scale values) within the reconstructed image and ED of the material being imaged is necessary. This is known as a MV CBCT ED calibration curve and is required, as it provides the treatment planning dose calculation algorithms with the required density information. The use of a calibration curve either relating ED and MV CBCT numbers directly^17^, ^19^ or MV CBCT numbers with kV CT numbers^(13)^ which are then correlated to ED, is noted in a number of recent publications utilizing MV CBCT images for radiotherapy treatment planning purposes.

Due to the insignificance of the photoelectric effect at MV energies, the MV CBCT ED calibration curve is expected to be linear,^(18)^ and the Feldkamp CB algorithm^(20)^ used to reconstruct the CBCT images makes this assumption.^(21)^ However, increased scatter from within the patient, the nonmonoenergetic nature of the beam, and the energy response of the electronic portal imaging device (EPID), all affect this linear relationship and need to be accounted for.^(21)^ One known result of the nonmonoenergetic beam is beam hardening, which causes an increase in mean energy of the beam due to increased attenuation of low‐energy photons.^21^, ^22^ A number of studies have looked at correcting for these effects.^21^, ^23^, ^25^

A recent study has shown that kV CBCT numbers from the Elekta XVI system are highly dependent on the parameters selected for acquisition,^(26)^ with the use of CT–ED calibration curves specific to the corresponding acquisition parameters recommended for accurate dose calculation. Hatton et al.^(27)^ found that the addition of scattering material to the ED phantom to better represent a patient resulted in more accurate dose calculations when utilizing an on‐board imager on a Varian 2100iX linear accelerator. An investigation into the use of MV CT images for a helical tomotherapy unit also showed variation in the CT–ED calibration curves with material surrounding the phantom.^(18)^ However, the effect that the acquisition and reconstruction parameters have on the CT–ED calibration curve for the Siemens MV CBCT system has not been extensively studied, despite investigations into the use of this system for treatment planning purposes.^(17)^ This preclinical study aimed to investigate the effect the number of monitor units (MUs), the reconstruction filter, reconstruction matrix size, and the use of extra scattering material has on the MV CBCT ED calibration curve.

## II. MATERIALS AND METHODS

This study was conducted with a Siemens ONCOR ImpressionPlus linear accelerator (Siemens AG, Munich, Germany) incorporating the MVision MV CBCT system. A 6 MV radiation beam is used with a fixed field size of 274 mm×274 mm. This system utilizes an amorphous silicon (aSi) flat panel, OPTIVUE 1000ST (AG9‐ES, PerkinElmer Optoelectronics, Woodbridge, Ontario, Canada), which has an active detection area of $41\times 41{\;\text{cm}}^{2}$, 1024×1024 pixels, maximum acquisition rate of 7 frames per second, approximate readout time of 145 ms, and pixel pitch of 0.4 mm.

The MVision system acquires projection images as the gantry is rotated continuously clockwise through 200 degrees, from 270° to 110° (IEC convention^(28)^). The linear accelerator delivers a fraction of a MU at each arc increment which is used to acquire one frame of the MV CBCT image, and the gantry moves at a constant speed which is dependent on the number of MU utilized. The flat panel is at a fixed distance from the source of 145 cm. Projection images are corrected for differences in sensitivity of individual pixels before being used in the cone‐beam reconstruction using a filtered backprojection method based on the Feldkamp cone‐beam reconstruction algorithm.^(20)^

**Figure 1:**
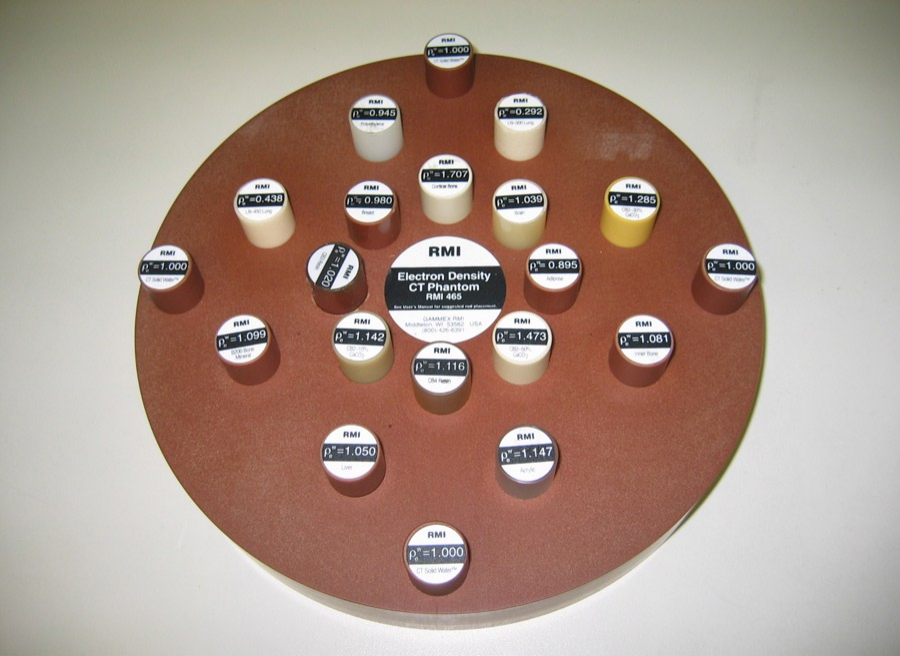
Electron density CT phantom.

A Gammex RMI (Gammex Inc., Middleton, WI) ED CT Phantom (Fig. 1), constructed of solid water and containing twenty holes fitted with interchangeable inserts of various tissue and water equivalent materials, was used. The insert's EDs range from 0.292 to 1.707 relative to the background solid water.

Images were acquired of the ED phantom, using parameters as detailed in Table 1. Four different MU settings were investigated. A smoothing reconstruction algorithm was utilized with either the Siemens ‘head and neck’ or ‘pelvis’ kernels. The use of extra scattering material to simulate the typical scattering conditions of a patient was also investigated. This was achieved by placing approximately $15\times 30\times 30\;{\text{cm}}^{3}$ solid water on either side of the phantom such that there was scattering material present in the whole cone beam field of view. Finally the effect of the matrix size of the reconstructed volume was investigated.

**Table 1:** MV CBCT parameters used for acquiring images of the electron density phantom.

*Monitor Units*	*Reconstruction Algorithm*	*Use of Scattering Material*	*Image Reconstruction Matrix Size*
5	H&N^a^	no	256×256×256 voxels
	H&N	no	“
8	pelvis	no	“
	pelvis	yes	“
	H&N	no	“
15	H&N	no	512×512×512 voxels
	H&N	yes	256×256×256 voxels
	pelvis	no	“
	H&N	no	“
60	pelvis	no	“
	pelvis	yes	“

^a^ H&N: head and neck

**Figure 2:**
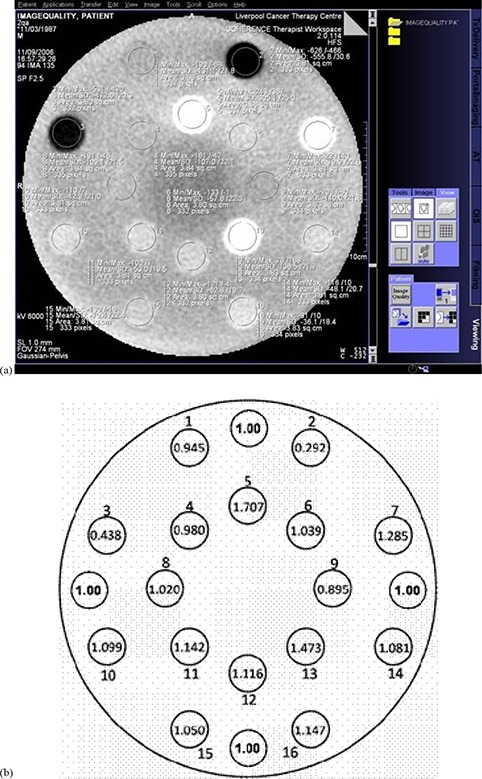
A MV CBCT image and diagram of the Gammex RMI electron density CT phantom with: (a) ROI for each insert marked on a reconstructed slice of the CT–ED phantom; and (b) location and ED of inserts including placement of water measurements (ED=1.00).

For each image set, a slice was chosen that was centrally placed within the phantom. On this slice, a circular region of interest (ROI) of approximately 2.5 cm diameter, slightly smaller than the insert itself, was drawn ((Fig. 2a). The arithmetic mean and standard deviation of pixel grey scale values were calculated for each insert. This process was averaged over five central slices to obtain an averaged result, such that the length of the ROI for the 256×256×256 reconstruction was approximately 5.5 mm, and for the 512×512×512 reconstruction was approximately 2.25 mm. Differences due to the difference in ROI size were assessed. It is noted that over the ROI and for surrounding slices, the density of the material imaged was constant. The solid water inserts were clipped out of the image due to the field of view. Therefore, results were obtained from the background solid water, as shown in (Fig. 2b).

The nonuniformity observed in the MV CBCT images due to beam hardening would have an impact on the MV CBCT number values obtained. To reduce this effect, the following geometric correction was applied after reconstruction. The percentage difference between the solid water pixel value at the position of the insert was determined by interpolating between the pixel values from immediately surrounding solid water and the average of five solid water pixel values around the phantom (four edges and centre), and this factor was applied to the results for all inserts, depending on their position. MV CBCT ED calibration curves were then generated for each image.

Comparisons of the resulting curves were undertaken to assess the change with MUs, the reconstruction algorithm, the presence of scattering material, and the size of the reconstructed image.

To assess the significance of possible dosimetric differences resulting from variations in the CT–ED calibration curves, dose calculations were compared for CT datasets simulated to represent the extremes of the measured CT–ED calibration curves. Simulated datasets were utilized to focus on dosimetric differences due to CT number rather than artifact in the image, which will vary from patient to patient. An in‐house software program was used to convert kV CT numbers to MV CBCT numbers for head and neck and for pelvis kV CT sets of an anthropomorphic phantom. The 15 MU head and neck reconstruction algorithm and pelvis reconstruction algorithm MV CBCT ED calibration curves were used, producing two simulated MV CBCT image sets representative of the head and neck, and two simulated MV CBCT image sets representative of the pelvis. Head and neck and cervix treatment plans were applied to the simulated MV CBCT image sets and the original kV CT image sets. Table 2 outlines the site, the kV CT to MV CBCT calibration curves used to simulate the MV CBCT image sets, and the CT to ED curve used within the treatment planning system to calculate dose. Dose calculations were undertaken with the XiO treatment planning system (CMS, St Louis, MO) using the superposition algorithm. This exercise was completed to illustrate the dose difference which would occur if the incorrect MV CBCT ED calibration curve was used. The difference in dose between the plans was calculated and dose‐volume histograms (DVHs) were compared for each plan.

**Table 2:** Outline of the image sets and CT to ED curves used within the treatment planning system to calculate the dose for the head and neck and pelvis treatment.

*Clinical Site and Treatment Plan*	*Image Set*	*CT‐ED Curve Used Within TPS to Calculate Dose*
Head and Neck Image and Treatment Plan	MV CBCT simulated with	MV CBCT ‘Head and Neck’
	kV CT to ‘Head and Neck’ MV CBCT curve	MV CBCT ‘Pelvis’
	MV CBCT simulated with kV CT to ‘Pelvis’	MV CBCT ‘Head and Neck’
	MV CBCT curve	MV CBCT ‘Pelvis’
	kV CT	kV CT
Pelvis Image with a Cervix Treatment Plan	MV CBCT simulated with kV CT to ‘Head and Neck’	MV CBCT ‘Head and Neck’
	MV CBCT curve	MV CBCT ‘Pelvis’
	MV CBCT simulated with kV CT to ‘Pelvis’	MV CBCT ‘Head and Neck’
	MV CBCT curve	MV CBCT ‘Pelvis’
	kV CT	kV CT

## III.RESULTS

### A. Monitor units

**Figure 3:**
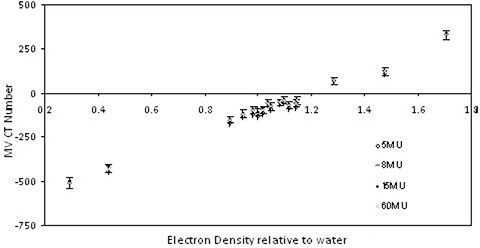
The MV CBCT ED calibration curves acquired with the head and neck 256 by 256 reconstruction kernel, no additional scattering material and 5 MU (o), 8 MU (−), 15 MU (+), and 60 MU (x).

Figure 3 shows the averaged mean CT number for each insert in the phantom, corrected for nonuniformity for the image sets acquired with the head and neck filter, 256 reconstruction kernels, and differing numbers of MUs.

**Figure 4:**
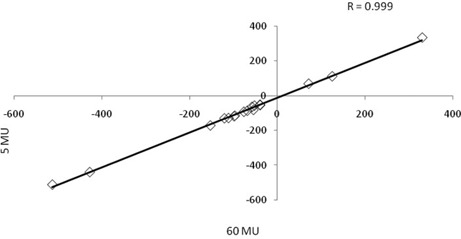
The correlation curve between MV CBCT numbers resulting from scans for 60 MU (x‐axis) and 5 MU (y‐axis). This shows a correlation coefficient between the two MU settings of 0.999.

The correlation coefficient between the 5 and 60 MU calibrations (Fig. 4) is 0.999, illustrating that the MV CBCT number is independent of the number of MU used during the calibration.

### B. Smoothing kernel

**Figure 5:**
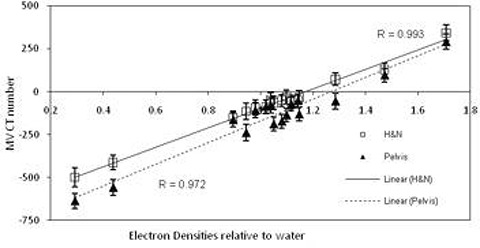
ED relative to water against resulting MV CBCT numbers, that is the MV CBCT ED calibration curves and the linear correlation coefficient values, for scans undertaken with the Gammex ED phantom using the pelvis (triangle markers) and head and neck (square markers) reconstruction kernels and 15 MU.

Figure 5 illustrates the lack of correlation between image sets when two different kernels were utilized during the reconstruction process with 15 MU. Reconstructed MV CBCT numbers were lower with the pelvis kernel. Similar results were seen with 8 and 60 MU.

### C. Matrix size

**Figure 6:**
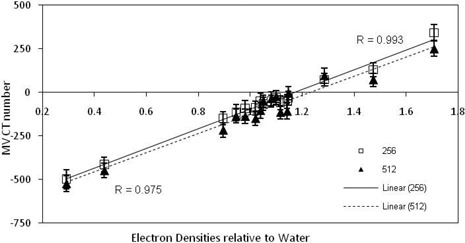
ED relative to water against resulting MV CBCT numbers, that is the MV CBCT ED calibration curves and the linear correlation coefficient values, for scans undertaken with the Gammex ED phantom using matrix sizes of 256×256×256 (square markers) and 512×512×512 (triangle markers) with the head and neck reconstruction kernel and 15 MU.

As illustrated in Fig. 6, the MV CBCT ED calibration curve was seen to be dependent on the matrix size used in the reconstruction. For the materials investigated, the increase in matrix size causes a reduction in CT number. The difference in uncertainty due to the difference in the ROI used for the 256 and 512 images was less than 1% and thus did not impact on the size of the difference in the calibration curves seen.

### D. Extra scattering material

**Figure 7:**
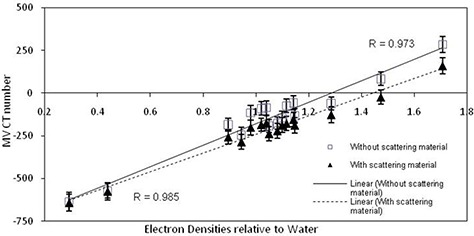
ED relative to water against resulting MV CBCT numbers, that is the MV CBCT ED calibration curves and the linear correlation coefficient values, for scans undertaken with the Gammex ED phantom without scattering material (square markers) and with scattering material (triangle markers) with the pelvis reconstruction kernel and 60 MU.

**Figure 8:**
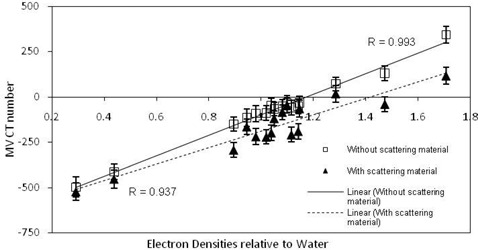
ED relative to water against resulting MV CBCT numbers, that is the MV CBCT ED calibration curves and the linear correlation coefficient values, for scans undertaken with the Gammex ED phantom without scattering material (square markers) and with scattering material (triangle markers) with the head and neck reconstruction kernel and 15 MU.

The addition of extra scattering material is illustrated in Figs. 7 and 8 for both the head and neck and the pelvis kernels.

In both cases, a reduction in the MV CBCT number can be seen when scattering material was used over the range of EDs investigated. This result was also evident at other MU settings.

The parameter showing the greatest influence on maximum variation or range in MV CBCT number across all parameter variations and all ED samples was the presence of scattering material, with the greatest difference seen for lower MU with the pelvis algorithm (Table 3).

**Table 3:** Mean and range of variation in MV CBCT number across the ED samples considered for each of the parameters varied.

*Parameter Varied*	*Mean MV CBCT Number*	*MV CBCT Maximum Variation*
Monitor Units	19.86	29.43
Reconstruction Kernel	70.74	144.65
Reconstruction Size	41.52	93.35
Surrounding Solid Water; 60 MU	67.54	124.91
Surrounding Solid Water; 15 MU	96.27	227.12

The maximum variation in MV CBCT number for the different EDs across all parameters was greatest for the highest ED and lowest MU setting (Table 4). The increase in maximum variation was not linear with ED.

**Table 4:** Mean and range in MV CBCT number across all parameters considered (reconstruction kernel and size, presence or absence of scattering material) varied according to ED and MUs.

	*15 MU (pelvis algorithm)*		*8 MU (pelvis algorithm)*		*60 MU (head and neck algorithm)*	
*Insert ED*	*Mean MV CBCT Number*	*Maximum Variation*	*Mean MV CBCT Number*	*Maximum Variation*	*Mean MV CBCT Number*	*Maximum Variation*
0.292	−544.98	134.24	−603.35	134.07	−597.08	129.78
0.438	−468.07	144.65	−532.17	173.46	−525.36	148.33
0.895	−205.42	143.76	−198.15	99.65	−198.67	104.68
0.945	−163.31	122.24	−221.28	164.01	−217.82	165.45
0.98	−138.80	128.33	−145.30	111.96	−138.41	102.00
1.02	−136.13	135.24	−122.63	104.09	−124.19	94.61
1.039	−107.36	150.56	−106.37	116.58	−106.70	117.99
1.05	−105.77	136.64	−169.12	169.82	−169.45	161.96
1.081	−85.27	135.32	−152.07	167.04	−150.04	168.51
1.099	−58.58	111.31	−126.16	145.32	−123.99	148.25
1.116	−113.52	149.17	−108.91	125.44	−106.55	108.78
1.142	−100.22	138.97	−94.99	103.14	−89.04	100.41
1.147	−58.25	124.47	−117.14	147.82	−119.91	148.58
1.285	33.05	149.17	−39.41	196.40	−38.60	197.68
1.473	64.71	169.72	66.20	144.90	61.48	148.85
1.707	250.30	227.12	261.11	176.54	258.13	170.11

**Figure 9:**
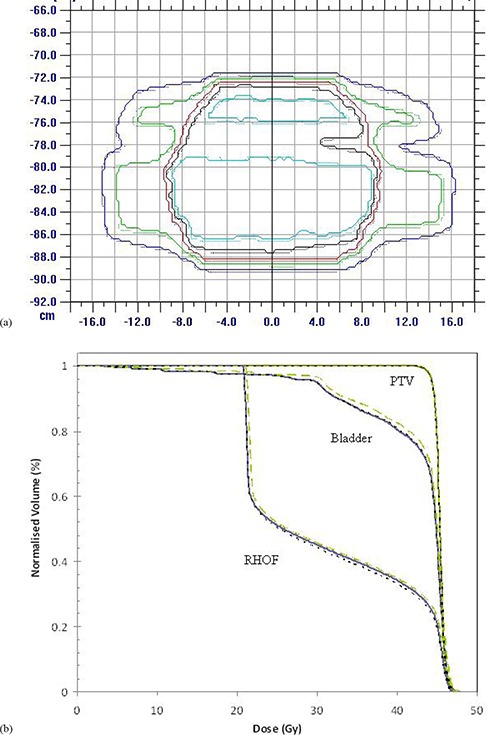
A comparison of cervix treatment plans with i) the original kV electron density information with a kV electron density calibration curve; ii) a simulated image representative of head and neck MV CBCT parameters with an electron density calibration curve from the same head and neck MV CBCT parameters; and iii) a simulated image representative of head and neck MV CBCT parameters with an electron density calibration curve from pelvis MV CBCT parameters. An isodose comparison (a) between plan ii (solid lines) and plan iii (dashed lines) for 10, 20, 30, 40, and 45 Gy (external to internal) isodose lines. Dose volume histograms (b) for the planning target volume (PTV), bladder, and right head of femur (RHOF) for plan i (small line dashes), plan ii (solid lines), and plan iii (large line dashes).

Small differences in the isodose lines with the MV CBCT data based on simulated head and neck parameters when both the head and neck and pelvis parameter electron calibration curves were used are seen in (Fig. 9a). Similarly, small differences in the DVH curves, due to the selection of the electron density calibration curve for the same dataset, are seen in (Fig. 9b). Differences in both the absolute dose and the DVH curves for cervix plans with the MV CBCT data based on simulated pelvis parameters (not shown) demonstrated differences of equivalent magnitude to the MV CBCT data based on simulated head and neck parameters. The largest differences were seen for the critical structures for the cervix treatment plan. Similar small differences were seen for the head and neck treatment plan with changes in the MV CBCT‐simulated image and the MV CBCT electron density calibration curve.

## IV. DISCUSSION

This study found that the number of MU used in the ED calibration does not dramatically alter the MV CBCT numbers obtained for different ED materials (Fig. 3). However, a change in reconstruction kernel, size of the reconstruction matrix, and presence or absence of scattering material does alter the MV CBCT calibration. Recent publications utilizing MV CBCT for dose calculations assume a linear relationship^(17)^ or present a linear relationship^13^, ^16^ that is utilized, but do not present the consistency of the linear relationship between different parameters.

Our study indicates that any MU setting between 5 and 60 MU could be used for the MV CBCT ED calibration curve. Several papers have presented MV CT–ED calibration^8^, ^18^, ^23^, ^29^, ^30^ for Tomotherapy, as well as linac‐based systems. Ruchala et al.^(29)^ report an “almost perfect” correlation (0.999) between the results obtained at a high dose and a more clinically relevant 7 cGy. Similarly, our study illustrates a correlation between the 5 and 60 MU calibrations of 0.999. Recent publications utilizing MV CBCT for dose calculations do not discuss differences in MU between the calibration curve and clinical or phantom images on which dose calculations are considered.^13^, ^16^, ^17^ Morin et al.,^(19)^ however, have demonstrated a change in contrast to noise ratio, due to change in CT number, with changing MU.

The results for the reconstruction smoothing kernel and reconstruction matrix size both demonstrated a noticeable change in MV CBCT number when these parameters were changed (Figs. 5 and 6). As noted by in the Morin study, binning, averaging, and diffusion filtering on the raw projections influences the MV CBCT numbers and the resulting signal‐to‐noise ratios. This variation in MV CBCT number suggests that a dose calculation error could result should a different smoothing kernel or reconstruction matrix size be utilized for a scan than was used for the MV CBCT ED calibration. Recent studies investigating the effect of altering acquisition parameters on kV CBCT have found that dose calculation errors can result.^26^, ^31^

The addition of scattering material to better represent a patient given the field sizes used for MV CBCT resulted in the greatest change in MV CBCT number (Figs. 7 and 8 and Table 3). This difference occurs due to the increased scatter that results in the considered slices from material outside this slice when scattering material is present, compared to the situation when this material is not present. This is particularly important for large field cone‐beam images, such as those considered here, as scattered radiation can result from material anywhere within this field. For fan‐beam CT systems, the addition of material outside the central volume does not result in the same impact, due to the small field size in the slice direction. This has also been reported for a kV CBCT system.^(27)^ The results of our study suggest that a similar approach to that suggested for kV CBCT systems,^(26,32)^ utilizing both site and parameter specific CT–ED calibration curves generated under full scatter conditions, should be utilized for MV CBCT images used in treatment planning. A larger phantom could be developed that is more appropriate for MV CBCT ED calibration. Previous studies, although utilizing MV CBCT ED calibration curves for dose calculations^13^, ^17^, ^19^ and considering the variation in image quality with change in parameter settings,^(19)^ have not considered the variation in MV CBCT ED calibration curve with changes in parameter settings. Although not assessing MV CBCT ED calibration curve variation, Morin et al.^(19)^ showed changes in contrast to noise ratio with variation in reconstruction protocols, possibly also influencing the MV CBCT ED calibration.

When determining CT–ED calibration curves, different numbers of material densities have been used by different investigations. Petit et al.^17^, ^21^ utilized two densities, that of water and air. Thomas et al.^(13)^ utilized three densities — air, bone, and tissue. This study, and that of Langen et al.^(18)^ and Cheung et al.,^(16)^ used up to 16 densities. It is noted that although the measured CT–ED calibration curve for MV CBCT was shown in this and other studies to be a linear relationship, as expected theoretically, there are uncertainties which should be understood and may not be detected with a limited number of material densities. The variation in MV CBCT numbers with different parameters was greatest for the highest electron density considered; however, the effect was not linear with electron density, likely due to beam hardening effects for the different densities.

The variation in MV CBCT ED calibration curves presented in this work suggested that site and parameter specific CT–ED calibration curves could be necessary to ensure accurate dose calculations. In the investigations undertaken for this study, the cupping artifact was corrected for with a geometrical approach, utilizing the ED of water at different positions within the phantom to correct the density at each of the plug positions. It is noted that this approach does not correct the ED for all pixels in the image, as undertaken with previously published approaches.^(21)^ For this reason, the difference in impact on dose calculations between the different calibration curves was considered with simulated images, representing the difference in CT number but not the cupping artifact. The difference in resulting dose distributions when using different MV CBCT CT ED calibration curves was small and unlikely to result in any difference to treatment in a clinical situation. This correlates well with previous work^26^, ^31^ undertaken for different cone‐beam systems, which also showed small differences when cone‐beam images were used for dose calculations. It is noted, however, that this error would add to other uncertainties already present in the reported dose. The most accurate MV CBCT ED calibration curve will be that generated with the same acquisition and reconstruction parameters as the patient dataset considered and under similar scatter conditions.

## V. CONCLUSIONS

This investigation has highlighted several factors that affect the MV CBCT ED calibration curve determined for the Siemens megavoltage MVision system. Changes in both the reconstruction smoothing kernel and reconstruction matrix size resulted in changes in MV CBCT numbers, as did the addition of scattering material around the standard ED phantom. Treatment planning calculations showed the use of a nonparameter specific MV CBCT ED calibration curve is unlikely to result in any clinical differences. However, to ensure minimal uncertainties in dose reporting, MV CBCT ED calibration measurements could be carried out using parameter‐specific calibration measurements, as has already been proposed for kV CBCT images.
